# Interventions to improve health care provider implementation and patient adherence of patients to recommendations on geriatric assessment and management in older adults: A scoping review protocol

**DOI:** 10.1371/journal.pone.0317177

**Published:** 2025-01-24

**Authors:** Laura Freeman, Ainslee Smith, Nai-Wen Ku, Ana Patricia Ayala, Fay Bennie, Arielle Berger, Tyler R. Chesney, Ploa Desforges, Kristen Haase, Jennifer Jones, Annie Lacerte, Caroline Mariano, Rajin Mehta, Ines Menjak, Johanne Monette, Richard Norman, Nicholas Legacy, Maryjo Antonio, Eric Pitters, Anne Stephens, Douglas Stephens, Doreen Wan-Chow-Wah, Camilla L. Wong, Shabbir M.H. Alibhai, Martine Puts

**Affiliations:** 1 Lawrence Bloomberg Faculty of Nursing, University of Toronto, Toronto, Ontario, Canada; 2 Gerstein Information Science Centre, University of Toronto, Toronto, Ontario, Canada; 3 Older Adult and Support Team Members, Toronto, Ontario, Canada,; 4 Toronto Grace Health Centre, and University of Toronto Department of Medicine, Toronto, Ontario, Canada; 5 Department of Surgery, Unity Health, Toronto, Ontario, Canada; 6 Department of Geriatrics, Université de Sherbrooke, Sherbrooke, Quebec, Canada; 7 UBC School of Nursing, University of British Columbia, Vancouver, British Columbia, Canada; 8 Department of Supportive Care, Princess Margaret Cancer Centre, University Health Network, Toronto, Ontario, Canada; 9 BC Cancer Agency, Vancouver, British Columbia, Canada; 10 Division of Geriatrics, Sunnybrook Health Sciences Centre, Toronto, Ontario, Canada; 11 Odette Cancer Centre, Sunnybrook Health Sciences Centre, Toronto, Ontario, Canada; 12 Division of Geriatric Medicine, Jewish General Hospital, Montreal, Quebec, Canada; 13 Division of Geriatric Medicine, McGill University Health Centre, Montreal, Quebec, Canada; 14 Department of Medicine, Unity Health, Toronto, Ontario, Canada; Universal Scientific Education and Research Network, UNITED KINGDOM OF GREAT BRITAIN AND NORTHERN IRELAND

## Abstract

The world population is aging. Comprehensive Geriatric assessment (CGA) has been proven to improve the well-being of older adults. However, evidence suggests not all clinicians implement these recommendations in their practice; nor do all patients adhere to them. Currently, there is no up-to-date review of interventions that can improve older adults’ adherence to CGA recommendations and health care provider/clinician implementation of those recommendations. The objective of this scoping review protocol is to describe the methodology of the scoping review with the aim to identify interventions that have been tested to improve adherence to CGA recommendations. We will use the Arksey and O’Malley framework and subsequent extension by Levac and colleagues to complete the scoping review. We searched OVID MEDLINE, OVID Embase, EBSCO CINAHL, APA PsychInfo, and Cochrane CENTRAL databases from inception to November 14, 2024, and will include a review of reference lists of included studies. Studies eligible for inclusion are studies of any design that examined one or more interventions aiming to improve clinician implementation of and patient adherence to CGA in any clinical setting. We will use standard methods for study selection, data abstraction, assessment of methodological quality of individual studies, and data synthesis. Results will be analyzed and reported using descriptive numerical summaries and narrative analysis. Findings from the scoping review will be published in a manuscript and presented at scientific conferences.

## Introduction

The world population is aging - the World Health Organization (WHO) estimates that by 2030 one in six persons in the world will be 60 years and over [1]. It is becoming increasingly important to address the needs of an aging population, thus the WHO recommends person-centered care that prioritizes the older adult’s needs and helps them achieve their life goals [2]. Comprehensive Geriatric Assessment (CGA) is a person-centered intervention that uses a holistic approach, which has been shown to improve the well-being of older adults [3–6].

CGA has not been widely implemented in many clinical settings thus far due to a lack of resources such as trained health care providers who have sufficient time to conduct CGA. For CGA to reach maximal benefit in improving health and quality of life, adherence of older adults receiving geriatric care to clinicians’ recommendations is crucial. Adherence to long-term therapies has been defined by the World Health Organization as “the extent to which a person’s behaviour- taking medication, following a diet, and/or executing lifestyle changes, corresponds with agreed recommendations from a health care provider” [7]. There has been only one narrative review reporting on rates of patient adherence to CGA in community-based clinics and the author reported that the older adults adherence rate to clinician recommendations ranged from 46 to 76% [8]. However, to the best of our knowledge there has not been a review of interventions aiming to improve patient adherence to CGA recommendations [8].

There is a need to better understand interventions aiming to improve the implementation of CGA recommendations by clinicians and patient adherence to those recommendations by older adults in clinical settings where the majority of patients are older adults and could therefore benefit from CGA. Furthermore, future studies that incorporate CGA as part of the clinical intervention could benefit from evidence on how CGA can be optimized in clinical settings where CGA is used. Therefore, the aim of this protocol article is to describe the methodology of a scoping review that will summarize interventions tested in any clinical setting to improve the implementation by health care providers and patient adherence to GCA. Scoping reviews are a form of knowledge synthesis that addresses exploratory research questions to map key concepts, types of evidence, and gaps within specific areas or fields [9]. Given our broad research question, a scoping review is appropriate and will best accomplish the aim of identifying and mapping interventions aimed at increasing implementation of CGA recommendations by clinicians and older adults’ adherence to CGA recommendations while exploring if there is sufficient evidence to support a future systematic review [10].

## Methods

We will follow the Arksey and O’Malley scoping review framework [11] and subsequent extension by Levac et al [12]. We will follow the PRISMA (Preferred Reporting Items for Systematic Reviews and Meta-Analyses) scoping review extension (PRISMA-ScR) [13] for reporting the scoping review (see S1 File: PRISMA-P Checklist). We developed this protocol using the Open Science Framework scoping review protocol guidelines [14].

### Eligibility criteria

We structured the inclusion criteria based on PICOSL (population, intervention, comparison, outcome, study design, language) elements.

- Population: older adults with any diagnosis from any country and any clinical setting, as well as their care partners and health care providers.
- Intervention: any intervention targeting clinicians’ implementation of and older patient adherence to CGA.
- Comparison: any comparator group/population if available.
- Outcome: Improvement of clinicians’ implementation of and older patient adherence to CGA.
- Study design: any type of peer-reviewed publication (full-text articles and conference abstracts) reporting on any type of study design (even case reports and case series) assessing interventions for improving clinician implementation and older patient adherence to CGA.
- Language: Studies published in English, French, Dutch or German because the research team can assess manuscripts in these languages.

We will exclude duplicate abstracts/full-texts, reviews, editorials, and commentaries.

### Information sources and search strategy

Our health sciences librarian (APA) completed a literature search using the following databases: OVID MEDLINE, OVID Embase, EBSCO CINAHL, APA PsycINFO and Cochrane CENTRAL from inception to November 14, 2024. The search was peer-reviewed using the Electronic Search Strategies (PRESS) checklist [15] by another librarian. We will supplement the search with a manual review of reference lists of included studies. The initial search was conducted using OVID MEDLINE please see Table 1 for the MEDLINE search strategy. All searches were translated to other databases using the appropriate controlled vocabulary. Please see S2 File Search strategies used November 14 2024 S1–S4 Tables in S2 File for the search strategies used for Embase, CINAHL, PsycInfo and Cochrane Central.

**Table 1:** Ovid MEDLINE(R) ALL 1946 to November 14, 2024 search strategy.

#	Searches	Results
1	Geriatric Assessment/	34018
2	(CGA or GAM).tw,kf.	8534
3	(geriatric^*^ adj2 assessment^*^).tw,kf.	7393
4	Health Services for the Aged/	18312
5	(age-friendly healthcare or age-friendly system^*^).tw,kf.	21
6	((frail^*^ or elder^*^ or senior^*^ or gerontolog^*^ or geriatric^*^ or veteran^*^ or old^*^ people old^*^ male^*^ or old^*^ female^*^ or old^*^ person^*^ or old^*^ resident^*^ or old^*^ adult^*^ or old^*^ patient^*^ or old^*^ wom?n or old^*^ men or old^*^ man or aged or sexagenarian^*^ or septuagenarian^*^ or octogenerian or octogenar^*^ or nonagenar^*^ or centenar^*^ or 80 year^*^ or 85 year^*^ or 90 year^*^ or 95 year^*^ or advanced age or super-aged or age over 80 or age over 85 or age over 90) adj3 (assess^*^ or evaluat^*^ or apprais^*^ or consultation^*^)).tw,kf.	31959
7	or/1-3 [GAM]	44418
8	“patient acceptance of health care”/ or patient compliance/ or “Treatment Adherence and Compliance”/	118877
9	((process^*^ or program^*^ or guideline^*^ or recommendation^*^ or plan or plans) adj3 (nonadher^*^ or adher^*^ or complianc^*^ or comply or complies or evaluat^*^ or uptake^*^ or uses or using or used or implement^*^ or uptaking)).tw,kf.	335252
10	(patient^*^ adj3 (complianc^*^ or comply or compliant)).tw,kf.	26520
11	implementation science/	1591
12	8 or 9 or 10 or 11	468889
13	(frail^*^ or elder^*^ or senior^*^ or gerontolog^*^ or geriatric^*^ or veteran^*^ or old^*^ people or old^*^ person^*^ or old^*^ resident^*^ or old^*^ adult^*^ or old^*^ patient^*^ or old^*^ wom?n or old^*^ men or old^*^ man or old^*^ female^*^ or old^*^ male^*^ or aged or sexagenarian^*^ or septuagenarian^*^ or octogenerian or octogenar^*^ or nonagenar^*^ or centenar^*^ or 80 year^*^ or 85 year^*^ or 90 year^*^ or 95 year^*^ or advanced age or super-aged or age over 80 or age over 85 or age over 90).tw,kf.	1982990
14	aged/ or “aged, 80 and over”/ or frail elderly/	3596830
15	4 or 5 or 13 or 14 [Older adults]	4855618
16	7 and 15 [Older adults and GAM]	38264
17	6 or 16 [GAM and older adults combined]	59229
18	12 and 17 [GAM AND Older adults AND compliance]	2176

### Study selection

We will first remove duplicate abstracts using Endnote then import the remaining abstracts into Covidence. In Covidence, we will use the deduplication function to further remove duplicates. We will use a two-step study selection process. First, two independent reviewers will read resultant titles and abstracts for eligibility using Covidence software. If there is no abstract but the title is relevant, it will be selected for full-text review. Irrelevant titles and titles and without abstracts will be excluded in the selection process. Disagreements will be resolved by a third reviewer using Covidence. Next, full texts of the resultant studies will be read and reviewed according to eligibility criteria by two independent reviewers. Disagreements during full-text selection will be resolved by a third reviewer. Articles not available at the University of Toronto library will be requested through the Interlibrary Loan system (https://onesearch.library.utoronto.ca/request-items-other-institutions-ill).

### Data charting and extraction

We will abstract data using a Microsoft Excel spreadsheet designed by LF and MP that includes our PICOSL elements. Data on general study characteristics (authors, publication year, title, journal, country, study design, randomization method, study population, study description, study results with regard to adherence and other outcomes) and intervention characteristics using the Template for Intervention Description and Replication (TIDieR) Checklist [16] will be collected by two independent reviewers.

Consistent with recommendations from Levac et al. [12], two reviewers will pilot test the data collection sheet during the initial data extraction process (an estimated two studies) to ensure it is consistent with the research question and purpose. We will resolve discrepancies between the two reviewers by consensus with a third reviewer. For any missing or unclear information, reviewers will e-mail the study authors to request or clarify information. If the authors do not reply within 2 weeks, a reminder email will be sent. If it is not possible to obtain the missing information from the study authors, data will be coded accordingly (“not reported”).

While a quality assessment of the included studies is optional for scoping reviews [13], we have chosen to include a quality assessment to be able to better summarize the available evidence identified in this review. The Mixed Methods Appraisal Tool (MMAT) version 2018 [17,18] uses the same quality assessment criteria for different types of study designs, which aligns well with this review where we will include all study designs that have been used to study an intervention to increase patient adherence to CGA. We will conduct quality assessment using the MMAT concurrently with the data abstraction for each study. Two reviewers will independently complete the quality assessment and disagreements will be resolved by consensus with a third reviewer. No study will be excluded based on the quality assessment.

### Summarizing, collating, and reporting results

We will follow the PRISMA scoping review guidelines [13] for reporting results and include a PRISMA flow diagram to graphically depict the study selection process. We will present the results of methodological quality assessment in a table. In accordance with principles of narrative synthesis, which is the mainstay of data synthesis in scoping reviews [10]), we will analyze and report extracted data based on their models of presentation in included primary studies.

## Discussion

CGA can improve well-being for older adults receiving care in clinical settings, however patient adherence to those recommendations is sub-optimal. We anticipate that even better outcomes can be achieved by CGA if patient adherence to clinical recommendations and health care provider/clinician implementation of CGA is optimized. To our knowledge, there has not been a systematic search for interventional studies aimed at improving patient adherence to CGA and health care provider/clinician implementation of CGA. The results of the scoping review will be relevant for clinicians using CGA in any setting, as there is a growing interest with the aging world population to use CGA effectively to improve outcomes for more vulnerable older adults [19].

This scoping review will not involve patients or the collection of primary research, therefore ethical approval is not required. Typically, as part of the Arksey and O’Malley scoping review framework, consultation with stakeholders is recommended [11]. As part of this review process, we will not complete a consultation as that will be done as part of the next study by our team.

Dissemination of the results of the scoping review to the broad scientific community will take place through the publication of a peer-reviewed article and presentation of results at scientific and clinical conferences.

A strength of our scoping review is a comprehensive search approach including multiple databases, multiple languages and searching for studies conducted in any clinical setting (specialized and non-specialized). A limitation is that some of the interventions that we will identify may be older and less relevant to the current clinical setting. It is important to understand what interventions have been tested so far so we can learn from previous studies. Another potential limitation is that we anticipate most studies will be from High Income Countries which will limit the generalizability of the findings to other parts of the world. Lastly, while CGA is an intervention for older adults, studies may be heterogeneous in terms of the older adult definition used across studies.

## Conclusion

This scoping review protocol provides an overview of the methodology of a scoping review that will provide a summary of the literature on interventions used to improve implementation by health care providers of and patient adherence to CGA recommendations when caring for older adults in any clinical setting. That information will be useful for health care providers to improve the implementation of CGA in their clinics.

Dr. Puts is the guarantor of the scoping review protocol.

## Supporting information

S1 File PRISMA-P Checklist. (DOCX)

S2 File Search strategy for all databases. (DOCX)
