# Supplementary material for: Interventions to improve health care provider implementation and patient adherence of patients to recommendations on geriatric assessment and management in older adults: A scoping review protocol
Source: PLoS One. 2025 Jan 24;20(1):e0317177. doi: 10.1371/journal.pone.0317177 (PMC11760611; doi:10.1371/journal.pone.0317177)
Supplement: S2 File — (DOCX) [file pone.0317177.s002.docx]

Supplemental file 2 Search strategies used November 14 2024.

Table S1 **Embase Classic+Embase** 1947 to 2024 November 14 Search Strategy

| **#** | **Searches** | **Results** |
| --- | --- | --- |
| 1 | geriatric assessment/ | 22876 |
| 2 | (CGA or GAM).tw,kf. | 12830 |
| 3 | (geriatric* adj2 assessment*).tw,kf. | 13081 |
| 4 | (age-friendly healthcare or age-friendly system*).tw,kf. | 41 |
| 5 | ((frail* or elder* or senior* or gerontolog* or geriatric* or veteran* or old* people old* male* or old* female* or old* person* or old* resident* or old* adult* or old* patient* or old* wom?n or old* men or old* man or aged or sexagenarian* or septuagenarian* or octogenerian or octogenar* or nonagenar* or centenar* or 80 year* or 85 year* or 90 year* or 95 year* or advanced age or super-aged or age over 80 or age over 85 or age over 90) adj3 (assess* or evaluat* or apprais* or consultation*)).tw,kf. | 49534 |
| 6 | or/1-3 [GAM] | 36801 |
| 7 | patient compliance/ | 164299 |
| 8 | ((process* or program* or guideline* or recommendation* or plan or plans) adj3 (nonadher* or adher* or complianc* or comply or complies or evaluat* or uptake* or uses or using or used or implement* or uptaking)).tw,kf. | 464237 |
| 9 | (patient* adj3 (complianc* or comply or compliant)).tw,kf. | 47316 |
| 10 | implementation science/ | 6884 |
| 11 | or/7-10 [Compliance] | 653114 |
| 12 | (frail* or elder* or senior* or gerontolog* or geriatric* or veteran* or old* people or old* person* or old* resident* or old* adult* or old* patient* or old* wom?n or old * men or old* man or old* female* or old* male* or aged or sexagenarian* or septuagenarian* or octogenerian or octogenar* or nonagenar* or centenar* or 80 year* or 85 year* or 90 year* or 95 year * or advanced age or super-aged or age over 80 or age over 85 or age over 90).tw,kf. | 2866260 |
| 13 | exp aged/ or exp aged hospital patient/ or exp frail elderly/ or exp institutionalized elderly/ or exp very elderly/ | 4164817 |
| 14 | 4 or 12 or 13 [Older adults] | 5949461 |
| 15 | 6 and 14 [Older adults AND GAM] | 28272 |
| 16 | 5 or 15 [GAM and Older adults combined] | 63033 |
| 17 | 11 and 16 [Older adults AND GAM AND Compliance] | 2474 |

Table S2 EBSCOhost interface CINAHL search strategy

| Fri, November 15, 2024 12:52:06 PM |
| --- |

| **#** | **Query** | **Limiters/Expanders** | **Last Run Via** | **Results** |
| --- | --- | --- | --- | --- |
| S18 | S12 AND S17 | Search modes - Proximity | Interface - EBSCOhost Research Databases Search Screen - Advanced Search Database - CINAHL Plus with Full Text | 1,651 |
| S17 | S7 OR S16 | Search modes - Proximity | Interface - EBSCOhost Research Databases Search Screen - Advanced Search Database - CINAHL Plus with Full Text | 33,554 |
| S16 | S4 AND S15 | Search modes - Proximity | Interface - EBSCOhost Research Databases Search Screen - Advanced Search Database - CINAHL Plus with Full Text | 19,462 |
| S15 | S13 OR S14 | Search modes - Proximity | Interface - EBSCOhost Research Databases Search Screen - Advanced Search Database - CINAHL Plus with Full Text | 1,254,641 |
| S14 | (MH "Aged, 80 and Over") OR (MH "Aged") | Search modes - Proximity | Interface - EBSCOhost Research Databases Search Screen - Advanced Search Database - CINAHL Plus with Full Text | 966,674 |
| S13 | TI ( frail* or elder* or senior* or gerontolog* or geriatric* or veteran* or old* people or old* person* or old* resident* or old* adult* or old* patient* or old* wom?n or old * men or old* man or old* female* or old* male* or aged or sexagenarian* or septuagenarian* or octogenerian or octogenar* or nonagenar* or centenar* or 80 year* or 85 year* or 90 year* or 95 year * or advanced age or super-aged or age over 80 or age over 85 or age over 90 ) OR AB ( frail* or elder* or senior* or gerontolog* or geriatric* or veteran* or old* people or old* person* or old* resident* or old* adult* or old* patient* or old* wom?n or old * men or old* man or old* female* or old* male* or aged or sexagenarian* or septuagenarian* or octogenerian or octogenar* or nonagenar* or centenar* or 80 year* or 85 year* or 90 year* or 95 year * or advanced age or super-aged or age over 80 or age over 85 or age over 90 ) | Search modes - Proximity | Interface - EBSCOhost Research Databases Search Screen - Advanced Search Database - CINAHL Plus with Full Text | 538,903 |
| S12 | S8 OR S9 OR S10 OR S11 | Search modes - Proximity | Interface - EBSCOhost Research Databases Search Screen - Advanced Search Database - CINAHL Plus with Full Text | 201,453 |
| S11 | (MH "Implementation Science") | Search modes - Proximity | Interface - EBSCOhost Research Databases Search Screen - Advanced Search Database - CINAHL Plus with Full Text | 1,581 |
| S10 | TI ( patient* N3 (complianc* or comply or compliant) ) OR AB ( patient* N3 (complianc* or comply or compliant) ) | Search modes - Proximity | Interface - EBSCOhost Research Databases Search Screen - Advanced Search Database - CINAHL Plus with Full Text | 8,120 |
| S9 | TI ( (process* or program* or guideline* or recommendation* or plan or plans) N3 (nonadher* or adher* or complianc* or comply or complies or evaluat* or uptake* or uses or using or used or implement* or uptaking) ) OR AB ( (process* or program* or guideline* or recommendation* or plan or plans) N3 (nonadher* or adher* or complianc* or comply or complies or evaluat* or uptake* or uses or using or used or implement* or uptaking) ) | Search modes - Proximity | Interface - EBSCOhost Research Databases Search Screen - Advanced Search Database - CINAHL Plus with Full Text | 162,570 |
| S8 | (MH "Patient Compliance") | Search modes - Proximity | Interface - EBSCOhost Research Databases Search Screen - Advanced Search Database - CINAHL Plus with Full Text | 36,315 |
| S7 | TI ( (frail* or elder* or senior* or gerontolog* or geriatric* or veteran* or old* people old* male* or old* female* or old* person* or old* resident* or old* adult* or old* patient* or old* wom?n or old* men or old* man or aged or sexagenarian* or septuagenarian* or octogenerian or octogenar* or nonagenar* or centenar* or 80 year* or 85 year* or 90 year* or 95 year* or advanced age or super-aged or age over 80 or age over 85 or age over 90) N3 (assess* or evaluat* or apprais* or consultation*) ) OR AB ( (frail* or elder* or senior* or gerontolog* or geriatric* or veteran* or old* people old* male* or old* female* or old* person* or old* resident* or old* adult* or old* patient* or old* wom?n or old* men or old* man or aged or sexagenarian* or septuagenarian* or octogenerian or octogenar* or nonagenar* or centenar* or 80 year* or 85 year* or 90 year* or 95 year* or advanced age or super-aged or age over 80 or age over 85 or age over 90) N3 (assess* or evaluat* or apprais* or consultation*) ) | Search modes - Proximity | Interface - EBSCOhost Research Databases Search Screen - Advanced Search Database - CINAHL Plus with Full Text | 19,282 |
| S6 | TI ( age-friendly healthcare or age-friendly system* ) OR AB ( age-friendly healthcare or age-friendly system* ) | Search modes - Proximity | Interface - EBSCOhost Research Databases Search Screen - Advanced Search Database - CINAHL Plus with Full Text | 9 |
| S5 | (MH "Health Services for Older Persons") | Search modes - Proximity | Interface - EBSCOhost Research Databases Search Screen - Advanced Search Database - CINAHL Plus with Full Text | 7,021 |
| S4 | S1 OR S2 OR S3 | Search modes - Proximity | Interface - EBSCOhost Research Databases Search Screen - Advanced Search Database - CINAHL Plus with Full Text | 20,683 |
| S3 | TI geriatric* N2 assessment* OR AB geriatric* N2 assessment* | Search modes - Proximity | Interface - EBSCOhost Research Databases Search Screen - Advanced Search Database - CINAHL Plus with Full Text | 3,213 |
| S2 | TI ( CGA or GAM ) OR AB ( CGA or GAM ) | Search modes - Proximity | Interface - EBSCOhost Research Databases Search Screen - Advanced Search Database - CINAHL Plus with Full Text | 1,600 |
| S1 | (MH "Geriatric Assessment+") | Search modes - Proximity | Interface - EBSCOhost Research Databases Search Screen - Advanced Search Database - CINAHL Plus with Full Text | 18,333 |

Table S3 **APA PsycInfo** 1806 to November 2024 Week 2 Search Strategy

| **#** | **Searches** | **Results** |
| --- | --- | --- |
| 1 | geriatric assessment/ | 1238 |
| 2 | (CGA or GAM).tw,id. | 513 |
| 3 | (geriatric* adj2 assessment*).tw,id. | 1276 |
| 4 | (age-friendly healthcare or age-friendly system*).tw,id. | 4 |
| 5 | ((frail* or elder* or senior* or gerontolog* or geriatric* or veteran* or old* people old* male* or old* female* or old* person* or old* resident* or old* adult* or old* patient* or old* wom?n or old* men or old* man or aged or sexagenarian* or septuagenarian* or octogenerian or octogenar* or nonagenar* or centenar* or 80 year* or 85 year* or 90 year* or 95 year* or advanced age or super-aged or age over 80 or age over 85 or age over 90) adj3 (assess* or evaluat* or apprais* or consultation*)).tw,id. | 9787 |
| 6 | treatment compliance/ | 18743 |
| 7 | ((process* or program* or guideline* or recommendation* or plan or plans) adj3 (nonadher* or adher* or complianc* or comply or complies or evaluat* or uptake* or uses or using or used or implement* or uptaking)).tw,id. | 112938 |
| 8 | (patient* adj3 (complianc* or comply or compliant)).tw,id. | 2889 |
| 9 | or/6-8 [Compliance] | 131442 |
| 10 | (frail* or elder* or senior* or gerontolog* or geriatric* or veteran* or old* people or old* person* or old* resident* or old* adult* or old* patient* or old* wom?n or old * men or old* man or old* female* or old* male* or aged or sexagenarian* or septuagenarian* or octogenerian or octogenar* or nonagenar* or centenar* or 80 year* or 85 year* or 90 year* or 95 year* or advanced age or super-aged or age over 80 or age over 85 or age over 90).tw,id. | 569393 |
| 11 | older adulthood/ | 21910 |
| 12 | 4 or 10 or 11 [Older adults] | 571665 |
| 13 | or/1-3 [CGA] | 2507 |
| 14 | 12 and 13 | 2092 |
| 15 | 5 or 14 | 10447 |
| 16 | 9 and 15 | 507 |

S4 Cochrane Central

Search Name: CGA Compliance Nov 15 2024 Scoping review

Date Run: 22/11/2024 23:12:29

| **ID** | **Searches** | **Results** |
| --- | --- | --- |
| 1 | MeSH descriptor: [Geriatric Assessment] this term only | 2093 |
| 2 | (CGA or GAM):ti,ab | 706 |
| 3 | (geriatric* NEAR/2 assessment*):ti,ab | 1158 |
| 4 | MeSH descriptor: [Health Services for the Aged] this term only | 581 |
| 5 | (age-friendly healthcare or age-friendly system*):ti,ab | 5 |
| 6 | ((frail* or elder* or senior* or gerontolog* or geriatric* or veteran* or old* people old* male* or old* female* or old* person* or old* resident* or old* adult* or old* patient* or old* wom?n or old* men or old* man or aged or sexagenarian* or septuagenarian* or octogenerian or octogenar* or nonagenar* or centenar* or 80 year* or 85 year* or 90 year* or 95 year* or advanced age or super-aged or age over 80 or age over 85 or age over 90) NEAR/3 (assess* or evaluat* or apprais* or consultation*)):ti,ab | 125556 |
| 7 | MeSH descriptor: [Patient Acceptance of Health Care] this term only | 4362 |
| 8 | MeSH descriptor: [Patient Compliance] this term only | 11904 |
| 9 | MeSH descriptor: [Treatment Adherence and Compliance] this term only | 201 |
| 10 | ((process* or program* or guideline* or recommendation* or plan or plans) NEAR/3 (nonadher* or adher* or complianc* or comply or complies or evaluat* or uptake* or uses or using or used or implement* or uptaking) ):ti,ab | 36095 |
| 11 | (patient* NEAR/3 (complianc* or comply or compliant)):ti,ab | 7851 |
| 12 | MeSH descriptor: [Implementation Science] this term | 108 |
| 13 | (frail*:ti,ab OR elder*:ti,ab OR senior*:ti,ab OR gerontolog*:ti,ab OR geriatric*:ti,ab OR veteran*:ti,ab OR (old* NEXT "people"):ti,ab OR (old* NEXT person*):ti,ab OR (old* NEXT resident*):ti,ab OR (old* NEXT adult*):ti,ab OR (old* NEXT patient*):ti,ab OR (old* NEXT wom?n):ti,ab OR (old* NEXT "men"):ti,ab OR (old* NEXT "man"):ti,ab OR (old* NEXT female*):ti,ab OR (old* NEXT male*):ti,ab OR aged:ti,ab OR sexagenarian*:ti,ab OR septuagenarian*:ti,ab OR octogenerian:ti,ab OR octogenar*:ti,ab OR nonagenar*:ti,ab OR centenar*:ti,ab OR 80 year*:ti,ab OR 85 year*:ti,ab OR 90 year*:ti,ab OR 95 year*:ti,ab OR "advanced age":ti,ab OR super-aged:ti,ab OR "age over 80":ti,ab OR "age over 85":ti,ab OR "age over 90":ti,ab) | 410766 |
| 14 | MeSH descriptor: [Aged] this term only | 279846 |
| 15 | #1 Or #2 Or #3 | 3342 |
| 16 | #4 OR #5 OR #13 OR #14 | 615018 |
| 17 | #15 AND #16 | 3042 |
| 18 | #17 OR #6 | 127119 |
| 19 | #7 OR #8 OR #9 OR #10 OR #11 OR #12 | 57995 |
| 20 | #18 AND #19 in Trials | 6197 |

**Total citations retrieved: 13,005**
